# Comparative analysis of computer-vision and BLE technology based indoor navigation systems for people with visual impairments

**DOI:** 10.1186/s12942-019-0193-9

**Published:** 2019-12-11

**Authors:** Jayakanth Kunhoth, AbdelGhani Karkar, Somaya Al-Maadeed, Asma Al-Attiyah

**Affiliations:** 1 Department of Computer Science and Engineering, Qatar University, Al Jamiaa Street, Doha, Qatar; 2 Department of Psychological Sciences, Qatar University, Al Jamiaa Street, Doha, Qatar

**Keywords:** Indoor navigation, People with visual impairments, Computer vision, Mobile technology

## Abstract

**Background:**

Considerable number of indoor navigation systems has been proposed to augment people with visual impairments (VI) about their surroundings. These systems leverage several technologies, such as computer-vision, Bluetooth low energy (BLE), and other techniques to estimate the position of a user in indoor areas. Computer-vision based systems use several techniques including matching pictures, classifying captured images, recognizing visual objects or visual markers. BLE based system utilizes BLE beacons attached in the indoor areas as the source of the radio frequency signal to localize the position of the user.

**Methods:**

In this paper, we examine the performance and usability of two computer-vision based systems and BLE-based system. The first system is computer-vision based system, called CamNav that uses a trained deep learning model to recognize locations, and the second system, called QRNav, that utilizes visual markers (QR codes) to determine locations. A field test with 10 blindfolded users has been conducted while using the three navigation systems.

**Results:**

The obtained results from navigation experiment and feedback from blindfolded users show that QRNav and CamNav system is more efficient than BLE based system in terms of accuracy and usability. The error occurred in BLE based application is more than 30% compared to computer vision based systems including CamNav and QRNav.

**Conclusions:**

The developed navigation systems are able to provide reliable assistance for the participants during real time experiments. Some of the participants took minimal external assistance while moving through the junctions in the corridor areas. Computer vision technology demonstrated its superiority over BLE technology in assistive systems for people with visual impairments.

## Introduction

Indoor navigation is becoming an important topic in the field of communication technology and robotics. It is the process of identifying the correct location of the user, planning a feasible path and ushering the user through the path to reach the desired destination [1]. The most important and challenging task in a navigation system is location estimation or localization of the user in real-time [2]. In outdoor environments, the location of the user is tracked using global positioning systems (GPS). GPS are not effective in indoor areas due to non-line of sight problem [3]. In order to overcome the limitations of GPS, various technologies are utilized to track the position of a user in the indoor environment [4]. Computer vision technologies [5] demonstrated their effectiveness in providing guidance for users. In the other hand, BLE-based navigation systems are still being developed and they are commercially available in the market [6]. In fact, BLE-based system are accurate but they require beacon devices to cover new areas with navigation services [7]. Based on this fact, we examine the performance and usability of a BLE-based navigation system in contrast with two computer-vision navigation systems.

In general, the computer-vision based systems utilize a smartphone or a device embedded with a camera such as a Google Glass to capture the scenes while the user is walking through the indoor areas. Localization using computer vision technology requires recognition of the visual scene by matching the captured picture with existing pictures or by employing trained models. The sort of these model differs depending on the targeted objectives, such as support vector machine (SVM) model [8], neural network model including deep learning model [9], and so. Apart from scene recognition some of the existing systems depend on recognizing the text or visual markers (QR codes or Barcodes) pasted in the indoor areas to provide reliable guidance for the people with VI.

BLE technology based systems make use of BLE beacons to estimate the location of the user. The BLE beacons are attached to the walls or ceilings of the indoor environments. A smartphone or a radio frequency signal receiver is used to record the RSS from the BLE beacons to estimate the position of the user. The most used position estimation techniques are lateration [10], BLE fingerprinting [11] and proximity sensing [12]. The lateration technique calculates the distance between the receiver device and the beacons. The proximity technique is based on the measurement of the proximity of the receiver to recently known locations. BLE fingerprinting applies a pattern matching procedure, where the RSS from a particular beacon will be compared with the RSS stored in the database.

In this work, we developed two computer-vision based navigation systems and a BLE based navigation system for people with VI in indoor areas. Moreover, we compared the performance of three systems which utilized three different approaches , convolution neural network (CNN) based indoor scene recognition, QR code recognition and BLE beacons based indoor positioning for guiding the people with VI in indoor areas. An indoor image data set has been built for indoor scene recognition application. We tuned the CNN model to recognize the indoor areas using the images in the dataset. This scene recognition model is extended for guiding people with VI.

All three systems are developed in a similar manner where a common android application is utilized for providing navigation aid to the users. The major difference between the three systems are the underlying positioning technique implemented on it. Moreover, except BLE based system, the rest are designed in a client-server architecture. The first computer vision based system namely ‘CamNav’ utilize indoor scene recognition technique to estimate the position of the user. CamNav employed a trained model to recognize the location from the query or captured images. CamNav use android application to capture the images and provide instruction to the user. The captured images are processed in the server using a trained deep learning model. The second computer-vision based system namely QRNav utilize visual markers called QR codes pasted in the indoor areas to determine the location of the user. Like CamNav, QRNav utilizes the Android application to capture the images and provide instruction to the user. The QR codes contained in the captured images are decoded by a state of art QR code decoder implemented in the server. The BLE based system consists of an Android application and an infrastructure made up of BLE beacons. Unlike computer-vision based systems, the BLE based system utilizes the Android application for users’ location estimation as well as providing instructions to the users. In addition to the development of the three navigation systems, the performance and usability of the systems are analyzed in real time environment. A field study with 10 blindfolded participants was conducted while using three systems. In order to compare and contrast the performance, efficiency, and merits of the three systems we considered navigation time, errors committed by the users and feedbacks from the users about the systems for further analysis. The remaining sections of the paper are organized as follows. In “Related work” section, we detail a fundamental study about the related work. In “Systems overview" section, we give an overview of CamNav, QRNav and BLE beacon based system. In “Evaluations and results” section, we present the evaluation, experimental setup and obtained results. In “Discussion” section, we discuss our findings. And finally, in “Conclusion” section, we conclude the paper.

## Related work

Considerable number of assistive systems, designed to augment visually impaired people with indoor navigation services, have been developed in the last decades. These systems utilize different types of technologies for guiding the people with VI in indoor areas. Since indoor positioning is the important task in navigation, in this segment initially we discuss various technologies and approaches utilized for positioning the user in indoor areas. Later we discuss and compare various existing navigation systems developed for people with VI.

### Overview of indoor positioning approaches

**Fig. 1:**
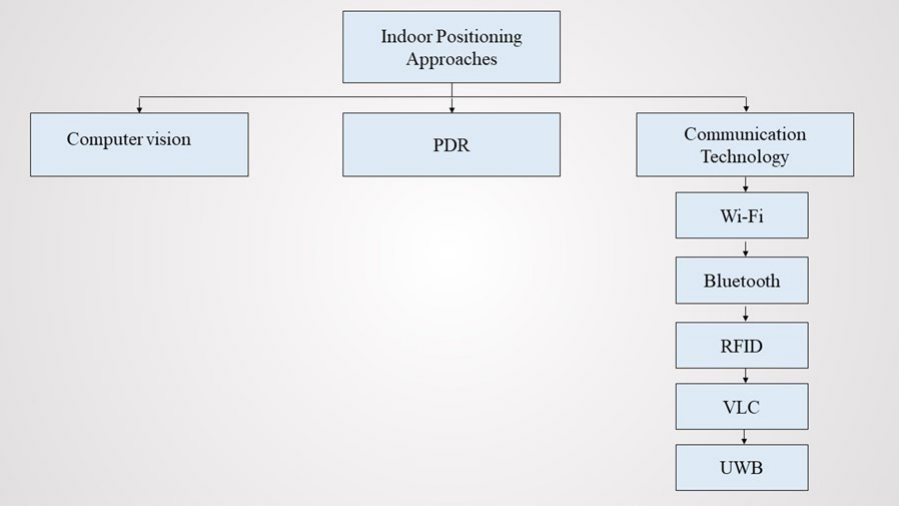
Hierarchical classification of indoor positioning approaches

Due to the inaccuracy of traditional GPS based approaches in indoor areas , high sensitive GPS and GPS pseudolites [13] are utilized for positioning the user in indoor areas. High sensitive GPS and GPS pseudolites displayed acceptable accuracy in indoor positioning, but their implementation cost is high. Apart from GPS based approaches, various technologies has been leveraged for the development of positioning module in indoor navigation systems. Figure 1 illustrates the hierarchical classification of common indoor positioning approaches utilized in indoor navigation systems.

Computer vision-based approaches make use of traditional cameras, omnidirectional cameras, 3d cameras or inbuilt smartphone cameras to extract visual imageries from the indoor environments. Diverse image processing algorithms such as Scale Invariant Feature Transform (SIFT) [14], Speeded Up Robust Feature (SURF) [15], Gist features [16] etc. were utilized for extracting the features from the captured imageries and followed by matching the query images. Together with the feature extraction methods, conventional approaches for vision-based positioning methods utilize clustering and matching algorithms. In addition to conventional approaches, deep learning-based computer vision solutions were developed in last 5 years. Deep learning models are composed of multiple processing layers to learn features of data without an explicit feature engineering process [17]. It made deep learning based approaches distinguished among object detection and classification methods. Apart from identifying the indoor locations based on matching the images, egomotion based position estimation methods were also employed in the computer-vision based positioning approaches [18]. Egomotion is the technique of estimating the positions of camera with respect to it’s surrounding environment.

Pedestrian dead reckoning (PDR) approaches estimate the position of the user based on their known past positions. PDR methods utilizes data acquired from the accelerometer, gyroscope and magnetometer to compute the position of the user. The traditional PDR algorithms compute the position of the user by integrating the step length of the user, number of steps traveled by the user and heading angle of the user [19, 20]. It is observed that conventional PDR approaches are abundant with position errors due to drift [21], varying step length of the users, sensor bias. In order to compensate the errors generated in traditional PDR approaches, most of latest PDR based systems combined another positioning technologies such as BLE or Wi-Fi along with it or introduced some novel sensor data fusion methodologies.

RFID, Wi-Fi, Ultra-Wide Band (UWB), Bluetooth and Visible Light Communication (VLC) are the popular communication technology based approaches utilized for indoor positioning task. RFID systems comprises of an RFID reader and RFID tags pasted on the objects. There exist two different types of RFID tags; active and passive. Passive tags does not require an external power supply. And many of the recent RFID based systems use passive RFID tags over active RFID tags. Time of arrival (TOA), Time difference of arrival (TDOA), Angle of arrival (AOA) and Received signal strength (RSS) the popular methods used in RFID based system for position estimation [22]. Indoor environment can contain different types of static objects such as walls, shelves etc. which can cause non-line of sight scenarios. In this context except RSS based position estimation, rest of the methods fails to compute the position of the user with minimal errors. The popular RSS based positioning approaches are trilateration and fingerprinting [23]. At present most of the indoor areas are equipped with Wi-Fi routers for providing seamless internet access for private groups or individuals or public groups. This existing Wi-Fi infrastructure can be utilized to localize the user and to provide navigation aid for the users. The Wi-Fi access points are used as the source for transmitting the signals to the receiving device ( mobile or small hardware) and receiving device utilize the received signal to estimate the position of the user. Despite the fact that Wi-Fi routers are costly compared to other RF signal transmitting devices, Wi-Fi based positioning methods displayed its popularity over other methods in recent years because of the availability of Wi-Fi routers in indoor areas. Wi-Fi-based indoor positioning systems make use of RSS fingerprinting or triangulation or trilateration methods for positioning [24]. Bluetooth based systems displayed similar or better accuracy in indoor positioning while comparing with Wi-Fi based systems. They make use of Bluetooth low energy (BLE) beacons installed in indoor environments to track the positions of users using lateration or proximity sensing approaches or RSSI fingerprinting [25]. In BLE systems , RSSI fingerprinting method has displayed better positioning accuracy while comparing with all other methodologies [26]. In order to preserve the efficiency of BLE indoor positioning systems, the data from the BLE beacons should be collected within a range of 3 meters. In recent advances, mostly a smartphone is used as a receiver for both Bluetooth and Wi-Fi signals.

Existing LED and florescent lamps in the indoor areas can be utilized for developing low cost indoor positioning solutions. Nowadays these LEDs or fluorescent lamps are becoming ubiquitous in indoor environment. Visible light communication (VLC) based indoor positioning methods use the light signals emitted by fluorescent lamps or LEDs to estimate the position of the user. A smartphone camera or dedicated independent photo detector is used to detect and receive the light signals from lamps. RSS and AOA are the most popular measuring approaches used in VLC based positioning methods [27]. UWB is also a radio technology which utilizes very low energy level for short-range, high-bandwidth communications. UWB based positioning systems can provide centimeter level accuracy which is far better than Wi-Fi based, or Bluetooth based methods. UWB uses TOA, AOA, TDOA, RSS based methodology for the position estimation [28].

Table 1 illustrates comparison of indoor positioning approaches

**Table 1:** Comparison of indoor positioning approaches

Indoor positioning technology	Infrastructure	Hardware	Popular measurement methods	Popular techniques	Accuracy
Computer vision	Dedicated infrastructure not required	Camera or inbuilt camera of smartphone	Pattern recognition	Scene analysis	Low to medium
Motion detection	Dedicated infrastructure not required	Inertial sensor or inbuilt sensors of smartphone	Tracking	Dead reckoning	Medium
Wi-Fi	Utilize existing infrastructure of building	Wi-Fi access points and smartphone	RSS	Fingerprinting and trilateration	Low to medium
Bluetooth	Dedicated infrastructure required	BLE beacons and smartphone	RSS and Proximity	Fingerprinting and trilateration	Medium
RFID	Dedicated infrastructure required	RFID tags and RFID tag readers	RSS and proximity	Fingerprinting	Medium
VLC	Dedicated infrastructure not required	LED lights and Photo detector	RSS and AOA	Trilateration and triangulation	Medium to high
UWB	Dedicated infrastructure required	UWB tags and UWB tag reader	TOA, TDOA	Trilateration	High

### Indoor navigation solutions for people with visual impairments

Computer vision is one of the popular technology used for the development of assistive systems for people with visual impairments. Computer vision based systems utilized two types of approach, tag based and non tag based approach to provide safe navigation for people with VI in indoor environments. Tag based system utilize some visual markers or codes attached in the indoor areas and tag or marker readers for guiding the user. Non tag based systems use the natural imageries of indoor areas or imageries of some static objects or texts found in the doors or walls in the indoor areas to guide the the people with VI in indoor areas. Moreover, 3 dimensional imageries are also utilized in the development of wayfinding or navigation system for people with VI.

Tian et al. [29] presented a navigation system for people with VI. It is composed of text recognition and door detection modules. The text recognition module employs the mean shift-based clustering for classifying the text, and Tesseract with Omni page optical character recognition (OCR) to identify the content of the text. The detection of doors is done by employing a canny edge detector. An indoor localization method has been proposed in [30]. It is based on the integration of edge detection mechanism with text recognition. Canny edge detector [31] is used to spot the edges in captured images. However, the usage of edge detection may fail in settings that have limited number of edges resulting in incorrect place recognition.

An android operating system based navigation system employ Google Glass to aid people with VI to navigate in indoor areas [32]. It uses canny edge detector and Hough line transform to detect the corners and object detection tasks. In order to estimate the distance from walls, a floor detection algorithm has been used. An indoor navigation system based on image subtraction method for spotting objects has been proposed in [33]. The algorithm of Histogram back-propagation is used for constructing the histograms of colors for detected objects. The tracking of the user is achieved by utilizing continues adaptive mean shift algorithm. A door detection method for helping people with VI to access unknown indoor areas was proposed in [34]. A miniature camera mounted on the head was used to acquire the scenes in front of the user. A computer module was included to process the captured images and provide audio feedback to the user. The door detection is based on a “ generic geometric door model” built on the stable edge and corner features. A computer vision module for helping blind peoples to access indoor environment was developed in [35]. An “image optimization algorithm” and a “two-layer disparity image segmentation” were used to detect the things or objects in indoor environments. The proposed approach examines the depth of information at 1 to 2 meters to guarantee the safe walking of the users.

Lee and Medioni proposed an RGB-D camera-based navigation system [36] for indoor navigation. Sparse features and dense point clouds are used to get the estimation of camera motions. In addition, a real-time Corner-based motion estimator algorithm was employed to estimate the position and orientation of objects which are in the navigation path. ISANA [37] consists of a Google tango mobile device, a smart cane with a keypad and two vibration motors to provide guided navigation for people with VI. The Google tango device is included with an RGB-D camera, a wide-angle camera, and 9 axes inertial measurement unit (IMU). The RGB-D camera was used to capture the depth data to identify the obstacles in the navigation path. The position of the user is estimated by merging visual data along with data from the IMU. Along with voice feedback, ISANA will provide haptic feedback to the user about the path and obstacles in noisy environments. The service offered by Microsoft Kinect device is extended to develop the assistive navigation system for people with VI [38]. The infrared sensor of Kinect device was integrated with RGB camera to assist the blind user. RGB camera is utilized to extract the imageries and infrared sensor is employed for getting the depth information to estimate the distance from obstacles. Corner detection algorithm was used to detect the obstacles.

A low cost assistive system for guiding blinds are proposed in [39]. The proposed system utilizes android phone and QR codes pasted in the floor to guide the user in indoor areas. ‘Zxing’ library was used for encoding and decoding the QR code. Zxing library for QR codes detection worked well under low light conditions. ‘Ebsar’ [40] utilizes a google glass, Android smartphone and QR codes attached to each location for guiding the visually impaired users. In addition to that, Ebsar utilizes the compass and accelerometer of the Android smartphone for tracking the movement of the user. The Ebsar provides instruction to the users in Arabic language. Gude et al. [41] developed an indoor navigation system for people with VI that utilizes bar code namely Semacode for identifying the location and guiding the user. The proposed system consists of two video cameras, one attached on the user’s cane to detect the tags in the ground and other one attached on the glass of the user to detect tags placed above the ground level. The system provides output to the user via a tactile Braille device mounted the cane.

The digital signs (tags) based on Data Matrix 2-D bar codes are utilized to guide the people with VI [42]. Each tag encodes a 16-bit hexadecimal number. To provide robust segmentation of tags from the surroundings, the 2D matrix is embedded in a unique circle-and-square background. To read the digital signs, a tag reader which comprises a camera, lenses, IR illuminator, a computer on module was utilized. etc. of. To enhance the salience of the tags for image capture, tags were printed on 3M infrared (IR) retro-reflective material. Zeb et al. [43] used fiducial markers (AR tool kit markers) printed on a paper and attached on the ceiling of each room for guiding the people with VI. Since the markers are pasted on the ceiling, the user has to walk with a web camera facing towards the ceiling. Up on successful marker recognition, the audio associated with the recognized marker stored in the database will be played to the user. A wearable system for locating blinds utilized custom colored markers to estimate the location of the user [44]. The prototype of the system consists of a wearable glass with a camera mounted on it and a mobile phone. In order to improve the detection time of markers, markers are designed as the QR code included inside a color circle. Along with these markers, multiple micro ultrasonic sensors were included in the system to detect the obstacles in the path of the user and thereby ensuring their safety. Rahul Raj et al. [45] proposed an indoor navigation system using QR codes and smartphone. The QR code is augmented with two information. First one is the location information which provides latitude, longitude, and altitude. The second one is the web URL for downloading the floor map with respect to the obtained location information. The authors address that usage of a smartphone with a low-quality camera for capturing the image and fast movement of user can reduce the QR code detection rate.

A smart handheld device [46] utilized visual codes attached to the indoor environment and data from smartphone sensors for assisting the blinds. The smartphone camera will capture the scenes in front of the user and search for visual codes in the images. Color pattern detection using HSV and YCbCr color space method were utilized for detecting the visual code or markers in the captured image. An Augmented Reality library called “ArUco is used as an encoding technique to construct the visual markers. Rituerto et al. [47] proposed a sign based indoor navigation system for people with visual impairments. The position of the user is estimated by combining data from inertial sensors of smartphone and detected markers. Moreover, the existing signs in indoor environments were also utilized for positioning the user. ArUco marker library was used to create the markers. The system will provide assistive information to the user via a text to speech module.

Nowadays BLE beacons based systems are becoming popular for indoor positioning and navigation application due to its low cost, and easiness to deploy as well as integrate with mobile devices. BLE beacons based systems are used in many airports, railways stations around the world for navigation and wayfinding applications. In the last 5 years, assistive systems developed for people with VI also relied on BLE technology to guide users in indoor areas. To best our knowledge there are few BLE beacons based indoor navigation systems proposed for people with VI. Most of the systems just require a smartphone and an Android or iOS application to provide reliable navigation service to the user. Lateration, RSSI fingerprinting and proximity sensing methods are the commonly used approaches for localizing the user.

NavCog [48] is a smartphone based navigation system for people with VI in indoor environment. The NavCog utilizes BLE beacons installed in the indoor environment and motion sensors of the smartphone to estimate the position of the user. RSSI fingerprint matching method was used for computing the position of user. Along with position estimation, the NavCog can provide information about the nearby point of interests, stairs, etc. to the users. StaNavi [49] is a similar system like NavCog, uses smartphone compass and BLE beacons to guide the people with VI. StaNavi was developed to operate in large railway stations. The BLE localization method utilized a proximity detection approach to compute the position of the user. A cloud-based server was also used in StaNavi to provide information about the navigation route. GuideBeacon [50] also uses smartphone compass and low-cost BLE beacons to assist the people with VI in indoor environment. GudieBeacon implemented a proximity detection algorithm to identify the nearest beacons and thereby estimate the position of the user. The system speaks out the navigation instructions using Google text to speech library. Along with audio feedback, the GuideBeacon provide haptic and tactile feedback to the user.

Bilgi et al. [51] proposed a navigation system for people with VI and hearing impairments in indoor area. The proposed prototype consists of BLE beacons attached on the ceiling of indoor areas and a smartphone. The localization algorithm uses nearness to beacons or proximity detection to compute the position of the user. Duarte et al. [52] proposed a system to guide the people with VI through public indoor areas. The system namely, SmartNav consist of a smartphone (Android application) and BLE beacons. The Google speech input API enables the user to give voice commands to the system. Android text-to-speech API is used for speech synthesis. The SmartNav utilizes multilateration approach to estimate the location of the user.

Murata et al. [53] developed a smartphone based blind localization system that utilize probabilistic localization algorithm to localize the user in a multi storied building. The proposed algorithm uses data from smartphone sensor and RSS of BLE beacons. Moreover, they introduced novel methods to monitor the integrity of localization in real world scenarios and to control the localization while traveling between floors using escalators or lift. RSS fingerprinting based localization in BLE beacons systems using fuzzy logic type 2 method displayed better precision and accuracy while compared to traditional RSS fingerprinting and other non fuzzy methods such as proximity, trilateration, centroid [54].

In addition to computer vision and BLE technology, RFID , Wi-Fi , PDR technologies are widely utilized for the development of assistive wayfinding or navigation systems for people with VI. Moreover, hybrid systems which integrate more than one technology to guide blinds or people with VI are proposed in the recent years. Table 2 illustrates Comparison of discussed indoor navigation solutions for people with visual impairments.

**Table 2:** Comparison of discussed indoor positioning solutions for people with visual impairments

References	Technology	System	Techniques	Tested by	Test	Feedback	Accuracy	Remarks
Tian et al. [29]	Computer vision	Web camera and mini laptop	Text recognition and door detection	Blinds	Door detection and door signage recognition	Voice	Medium	(−) Motion blur and very large occlusions happen when subjects have sudden head movements
Lee and Medioni [36]	Computer vision	RGB-D camera, IMU, Laptop	Camera motion estimation	Blinds and blindfolded	Pose estimation and mobility experiments	Tactile	Medium	(−) Inconsistency in maps
Manlises et al. [33]	Computer vision	Web camera and computer	CAMShift tracking	Blinds	Object recognition, navigation time	Voice	Medium	(−) Tested with 3 blinds only
Li et al. [37]	Computer vision	Tango mobile device	Obstacle detection, camera motion estimation by combining visual and inertial data	Blindfolded	Error occurred during navigation and navigation time	Voice and Haptic	Good	Using smart cane with the system reduced the errors
Kanwal et al. [38]	Computer vision	RGB camera and Kinect sensor	Corner detection using visual and inertial data	Blind and blindfolded	Obstacle avoidance and walking	Voice	Good	(−) Infrared sensor failed under strong sunlight conditions
Al-Khalifa et al. [40]	Computer vision and motion	Google glass, Android smartphone, QR code	QR code recognition, IMU	Blinds	Error occurred during navigation and navigation time	Voice	Medium	(+) Easy to use
Legge et al. [42]	Computer vision	Digital tags, Tag reader , smartphone	Digital tag recognition	Blinds and Blind folded	Tag detection, Route finding	Voice	Medium	(+) System provided independent navigation
Zeb et al. [43]	Computer vision	Web cam, AR markers	AR marker recognition	Blinds	Normal walking	Voice	Medium	Low cost
Ahmetovic et al. [48]	BLE	Beacons and smartphone	Fingerprinting, IMU	Blinds	Normal occurred events that hinder the navigation	Voice	Medium	(−) Can’t inform the users that they are in wrong way
Kim et al. [49]	BLE	Beacons and smartphone	Proximity detection, IMU	Blinds	Navigation time, task completion, deviation during navigation	Tactile and voice	Good	(+) Test was carried out in a large area (busy railway station)
Cheraghi et al. [50]	BLE	Beacons and smartphone	Proximity detection, IMU	Blinds	navigation time, Navigation distance	Haptic and voice	Medium	Improvements are required to support varying pace of walking

Many of the navigation systems discussed in this section have adopted several evaluation strategies to show their performance, effectiveness, and usability in real-time. Most of the systems considered navigation time as a parameter to show their efficiency. Some of the systems utilized the error committed by the users during navigation to asses the effectiveness of the system. The most important and commonly used approach is conducting surveys, interviews with the people who participated in the evaluation of the system. The user feedback help the authors to limit the usability issues of the proposed system.

## Systems overview

### CamNav

**Fig. 2:**
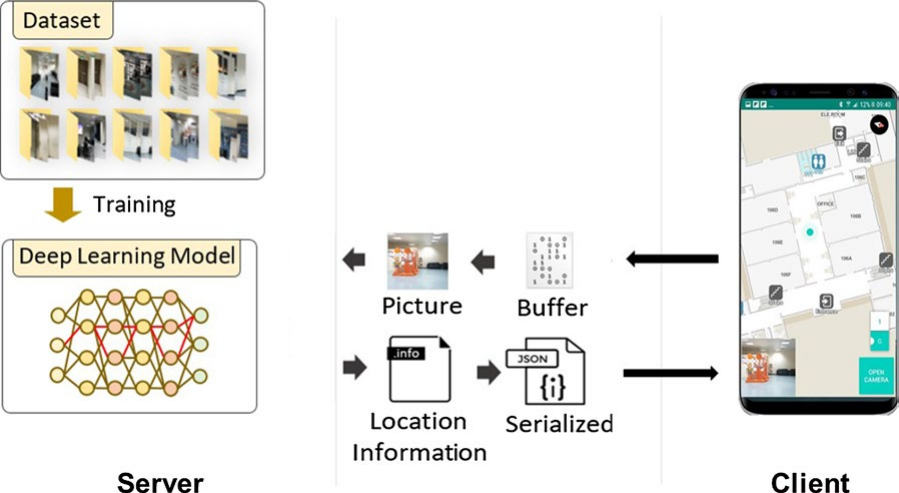
CamNav system overview

CamNav is a computer-vision based system, which utilizes a trained deep learning model to perform indoor scene recognition. Figure 2 shows an overview of the system. The architecture of the system is client-server.

#### Server application

The server application is responsible for processing the content of the queried images and identifying the location from the queried images. The server application utilizes a trained deep learning model to recognize the location from the image. The server application returns the location information to the mobile application in a JSON format. The server application utilizes an open source message broker namely, Active MQ [55] to communicate with the Android application.

#### Deep learning model

**Fig. 3:**
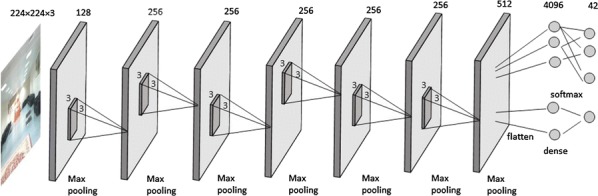
Architecture of the developed deep learning model

A deep learning model is configured for indoor scene recognition task. Tensorflow [56], an open-source machine learning library was utilized to build the deep learning model. Figure 3 illustrates the architecture of the developed deep learning model. The model is built using convolutional layers, pooling layers, and fully connected layers at the end.

For an input RGB image *i*, convolutional layer calculates output of the neurons which are associated to each local regions of the input. Convolutional layer can be applied to raw input data as well as output of another Convolutional layer. During convolution operation, the filter/kernel will slide over the each raw pixel of the RGB image or over the feature map generated from the previous layer. This operation compute the dot product between weights and regions of the input.

Let $M_i^{l-1}$ be the feature map from previous layer, $w_k^{l}$ is the weight matrix in current layer then convolutional operation will results new feature map $M_k^{l}$.

1 $$\begin{aligned}&Y= \displaystyle \sum \limits _{i\in N_{K}} M_i^{l-1}*w_k^{l}+b_k^{l} \end{aligned}$$

2 $$\begin{aligned}&M_k^{l}= f(Y) \end{aligned}$$

Here, *Y* is the output of convolutional operation. $N_{K}$ represents the number of kernel in current layer and $b_k^{l}$ is bais value. Bais is an additional parameter used in CNN to adjust the output from the convolutional layer. Bais help the model to fit best for input data.

An activation function *f(Y)* is applied to the resulting output from the convolutional operation to generate the feature map $M_k^{l}$. Activation function a.k.a transfer function is utilized to decide the output by mapping the resulting values of convolutional operation to a specific interval such as between [0,1] or [−1,1] etc. Here we utilized Rectifier Linear Unit (ReLU) as an activation function. ReLU is the commonly used activation function in CNN and faster compared to other functions.

For an input *x*, ReLU function *f(x)* is,

3 $$\begin{aligned} f(x) = {\left\{ \begin{array}{ll} 0, &{} \text {if }x < 0 \\ x, &{} \text {if }x \ge 0 \end{array}\right. } \end{aligned}$$

Once the convolutional operation is completed, pooling operation is applied on the resulting feature map to reduce the spatial size of feature maps by performing down sampling. Average pooling and max pooling are the two common functions utilized for pooling operation.

For a feature map of volume *W*1 $\times$ *H*1 $\times$ *D*1, pooling operation produce a feature map of reduced volume *W*2 $\times$ *H*2 $\times$ *D*2 where:

$$\begin{aligned} W2=(W1-F)/S+1, \quad H2=(H1-F)/S+1, \quad D2=N_{K} \end{aligned}$$

Here, *S* is stride and *F* is spatial extent. We used max pooling function in pooling operation where MAX operation is applied in a local region resulting a max value among that region.

The feature map in the form of n dimensional matrix are flattened in to vectors before feeding to fully connected layer. The fully connected layer combines the feature vector to build a model. Moreover, softmax function is used to normalize the output of fully connected layer that result the outputs representation based on probability distribution.

For an input image *x*, softmax function applied in the output layer computes the probability that x belongs to a class $c_{k}$ by,

4 $$\begin{aligned} p(y=c_{k}|x;P)= \frac{e^{P_{c_{k}}^T x}}{\displaystyle \sum \nolimits _{c_{i}=1}^n e^{P_{c_{i}}^T x}} \end{aligned}$$

where *n* is the number of classes and *P* is the parameter of the model.

Our model consist of 7 convolutional layers where each has a max pooling layer attached to it. The first convolutional layer has 128 filters with size $3\times 3$ and the last layer has 512 filters with size $3\times 3$. The other convolutional layers use 256 filters with size $3\times 3$. Max pooling layer is responsible for reducing the dimensions of the features obtained in its preceding convolutional layer. Dimensionality reduction aids the convolutional neural network model to achieve translation invariance, reduce computation and lower the number of parameters. In the end, the architecture contains a fully connected layer with 4096 nodes followed by an output layer with softmax activation. The deep learning model was trained using more than 5000 images to identify 42 indoor location. The model takes an RGB image of size $224\times 224$ pixels as input and classifies the image into one of the 42 class labels learned during the training phase.

#### Image dataset

**Fig. 4:**
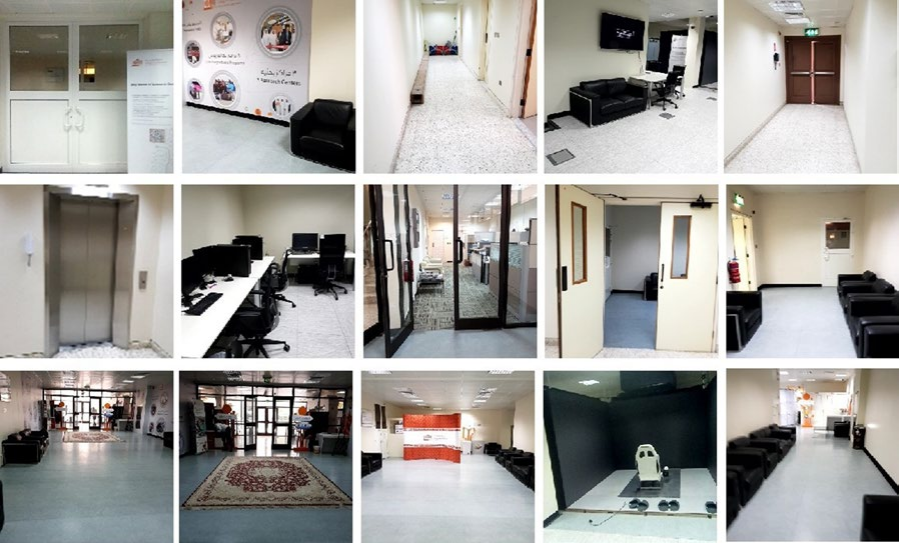
Sample images from dataset

Our indoor image dataset [57] contains more than 5000 images classified into different directories. Each directory represents one indoor location or class. Moreover, each directory contains a JSON file which contains the location information required by the Android application to locate the user. The images in the dataset are captured from the ground floor of building B09 of Qatar University. In order to consider various orientation of users, we captured pictures from different angles for the same location. The images are captured using Samsung Galaxy smartphone, LG smartphone and Lenovo smartphone. We considered the diversity of mobile phones to reserve the different sorts of pictures which are taken from varied cameras. Each images are in RGB format and reshaped into a size of $224 \times 224$ pixels. This dataset can be utilized for indoor scene recognition applications also. The sample images of the dataset are displayed in Fig. 4.

#### Navigation module

The navigation module is responsible for providing navigation instructions to the user. We utilized the indoor map and CAD drawings of indoor areas to create the navigation module. The navigation module contains the routing information between each point of interest to another point of interest. The navigation information inside the navigation module is stored in a JSON array format. One JSON array includes the navigation instructions for one specific route. We created the navigation instructions manually for each route.The common commands used in navigation instructions are turn right, turn left, walk straight etc. Moreover, instructions provide information such as distance between current location of the user (computed by the system) and critical locations such as junctions in indoor areas or doors or lift. The distance between each location associated with the captured visual scenes was measured manually and represented in terms of steps. For converting the distance measured in meters to step, we considered walking patterns of normal people.

#### Android application

**Fig. 5:**
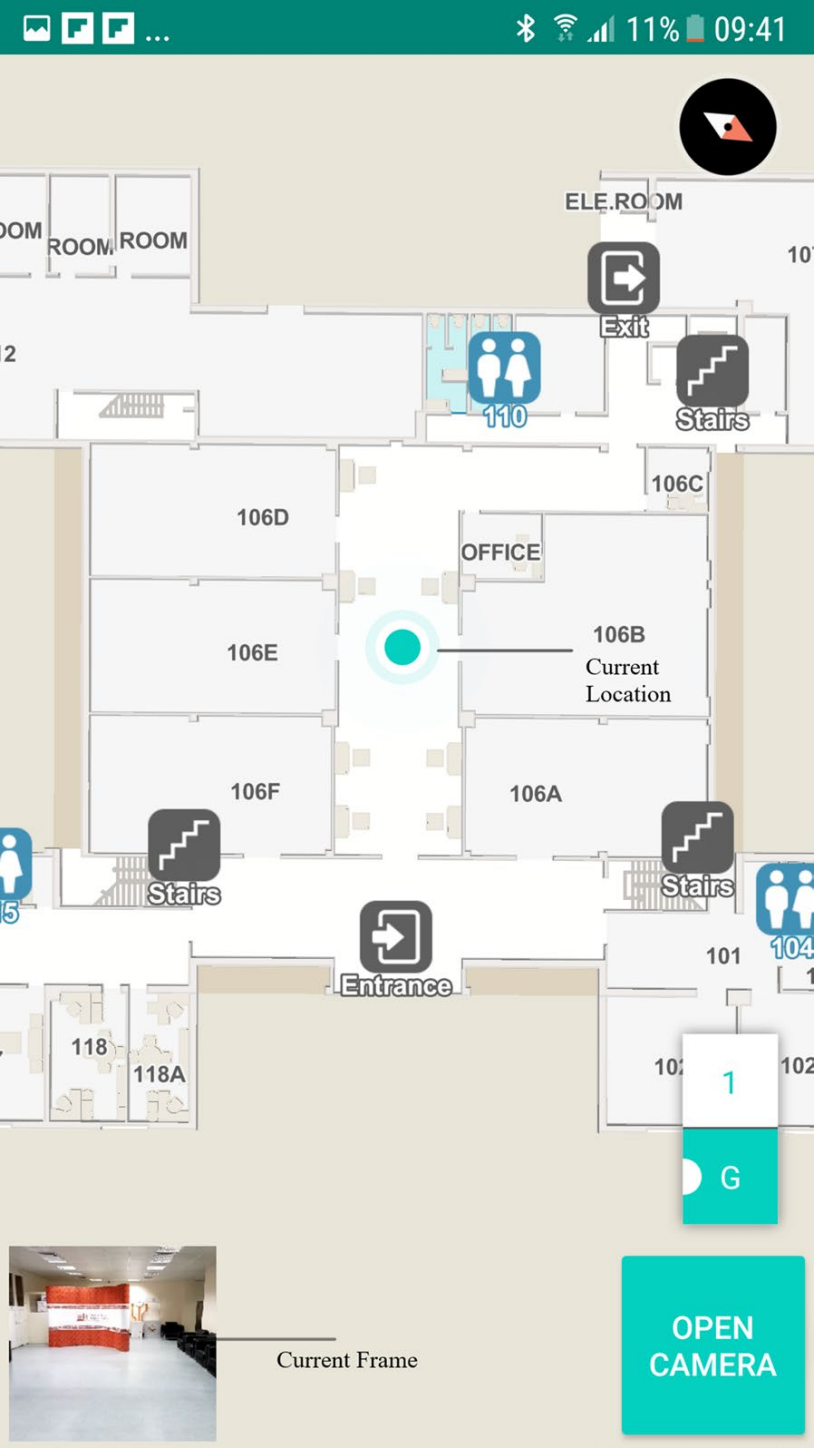
GUI of Android application

The android application captures the scenes in front of the user in a real-time manner for processing. The captured images are sent to the server application for further processing. The application contains an indoor map, a navigation module, and a speech to text module. The indoor map is created with [58]. The indoor map creation just requires computer-aided drawings of the building. The current location of the user has indicated with a turquoise color dot in the graphical interface of Android application as shown in Fig. 5. The turquoise color dot is a feature provided by Mapbox software development kit. The inputs required for the dot representation are the latitude, longitude, building number and building level. These data are made available in the directories associated with each location along with the images collected from that location in the indoor image dataset. Once the deep learning model classifies the query image, the JSON file in the directory associated with the output label will be sent to the Android application. The Android application serializes the received JSON file and updates the turquoise dot in the map. During navigation, the Android application will speak out the navigation and location information in regular intervals. A text to speech service is included inside the android application to provide voice instructions to the user. The text to speech service is developed using Android text to speech library. Android text to speech library is a popular and well accepted library used in the Android application development for the purpose of synthesizing the speech from text. Android text to speech library support many languages such as English , German, French, Japanese etc. The current version of the developed Android application can give voice instructions to the user in English language only. Visually impaired users can give instructions to the system using speech. We deployed a speech to text module in the application using Android inbuilt speech to text library for providing the mentioned service. People with VI can use this service for controlling various necessary functions of the system such as to start or end navigation, to select destination, to know the current location etc. Currently system can understand English language only.

### QRNav

**Fig. 6:**
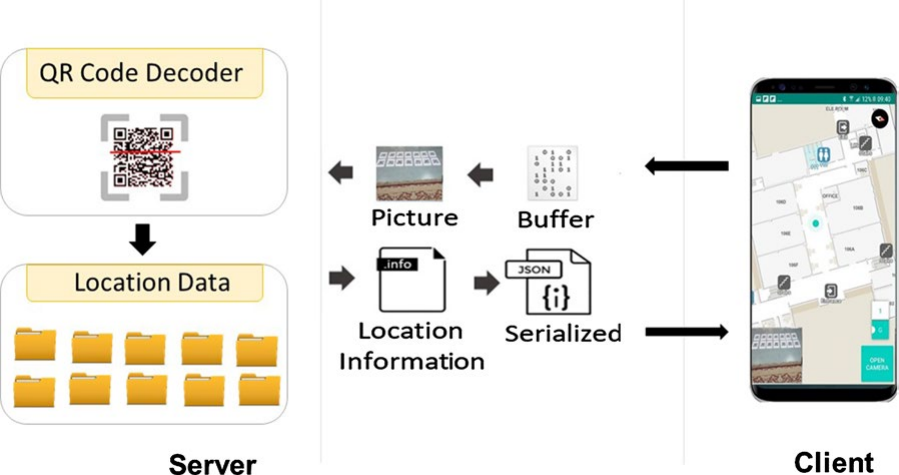
Overview of the QRNav system

QRNav system uses QR code recognition function to estimate the location of the user. Figure 6 depicts the overview of the QRNav system. The system consists of printed QR codes, QR code dataset, QR code decoder, and an Android mobile application.

The QR codes are attached on the ground, walls, and doors in indoor area of the building. The QR codes associated with each location have an embedded unique 4 digit id (encoded information). The android application captures the images while the user is walking. The captured images will be sent to the server. The server application contains QR code detection and decoding library to extract the unique id associated with the QR code. Once the server receives an image, the QR code detection method will look for QR codes in the image. If any QR code is detected, the decoding method will extract the unique id encoded QR code and return the location file (JSON file) associated with the unique id to Android application.

#### QR code decoder

We analyzed two open source barcode reader library, ‘Zxing’ [59] and ‘Zbar’[60] for QR code encoding and decoding operations. We found that ‘Zxing’ library is more effective compared to ‘Zbar’ in low light and challenging conditions. We implemented the ‘Zxing’ library and the ‘Zbar’ library to read and extract the information from QR codes.

#### QR dataset

**Fig. 7:**
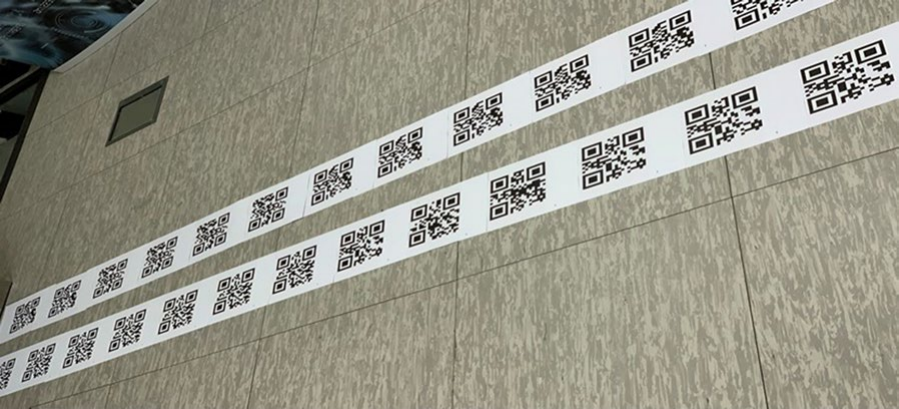
QR codes attached on the floor

The QR dataset contains more than 25 directories where each directory is associated with an indoor location. The ame of the directories are unique (same as unique id embedded in the QR codes). The directories are enclosed with a JSON file which contains information about the location. The QR codes are created using pyzbar library (Zbar library for python language). Each indoor location is mapped with a unique QR code. The four digits unique id beginning from ‘1000’ was manually assigned for each QR code. The QR code was printed in normal A4 sheet papers and pasted in indoor areas. In each location, we provided more than 25 copies of the QR code to provide reliable navigation service. Figure 7 shows the sample instance of attached QR codes on the floor.

#### Android application

The Android application contains the indoor map, text to speech module and navigation module. The text to speech module supports the speech synthesis and navigation module provides navigation instruction to the user. We utilized the same Android application used in CamNav system for QRNav also.

**Fig. 8:**
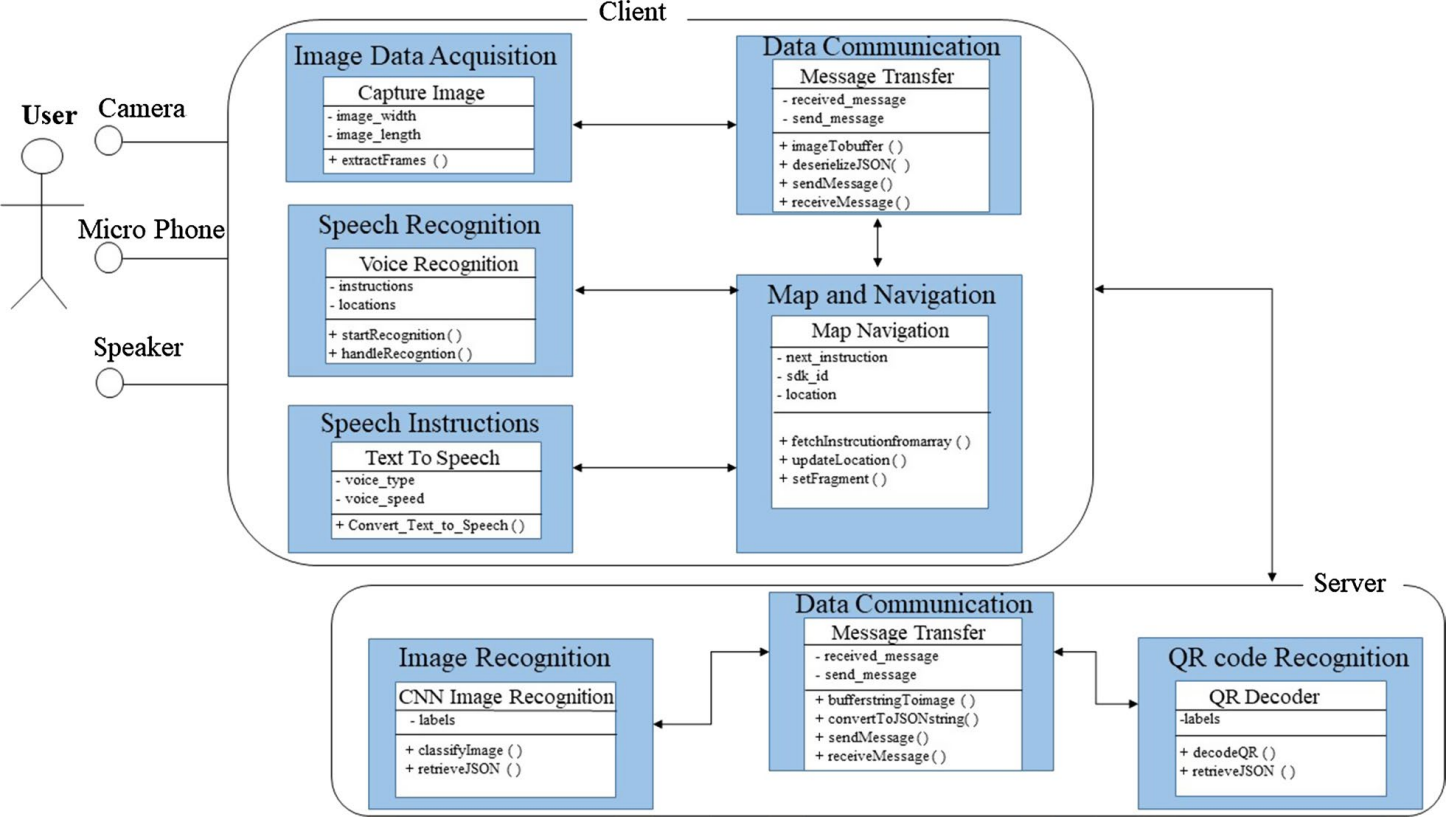
UML diagram of CamNav and QRNav

The functioning of both CamNav and QRNav are similar. Figure 8 depicts the UML diagram of both CamNav and QRNav system.

### BLE beacons based navigation

The Bluetooth low energy beacons based navigation system comprises of an Android application and BLE beacons fixed in the indoor environment. The BLE beacons based navigation system is developed with the help of indoor positioning SDK supplied by Steerpath.

#### BLE positioning module

The positioning module combined two popular positioning techniques to estimate the location of the user in real-time. BLE fingerprinting [11] and multilateration [61] techniques were utilized to achieve the localization of the user in the indoor map. When the system sense only two nearby beacons, then fingerprinting technique is utilized, where the observed fingerprint is compared with the pre-stored fingerprints in the database. If the system is able to detect more than two nearby beacons, the multilateration technique is used to compute the position of the user.

#### Beacons

**Fig. 9:**
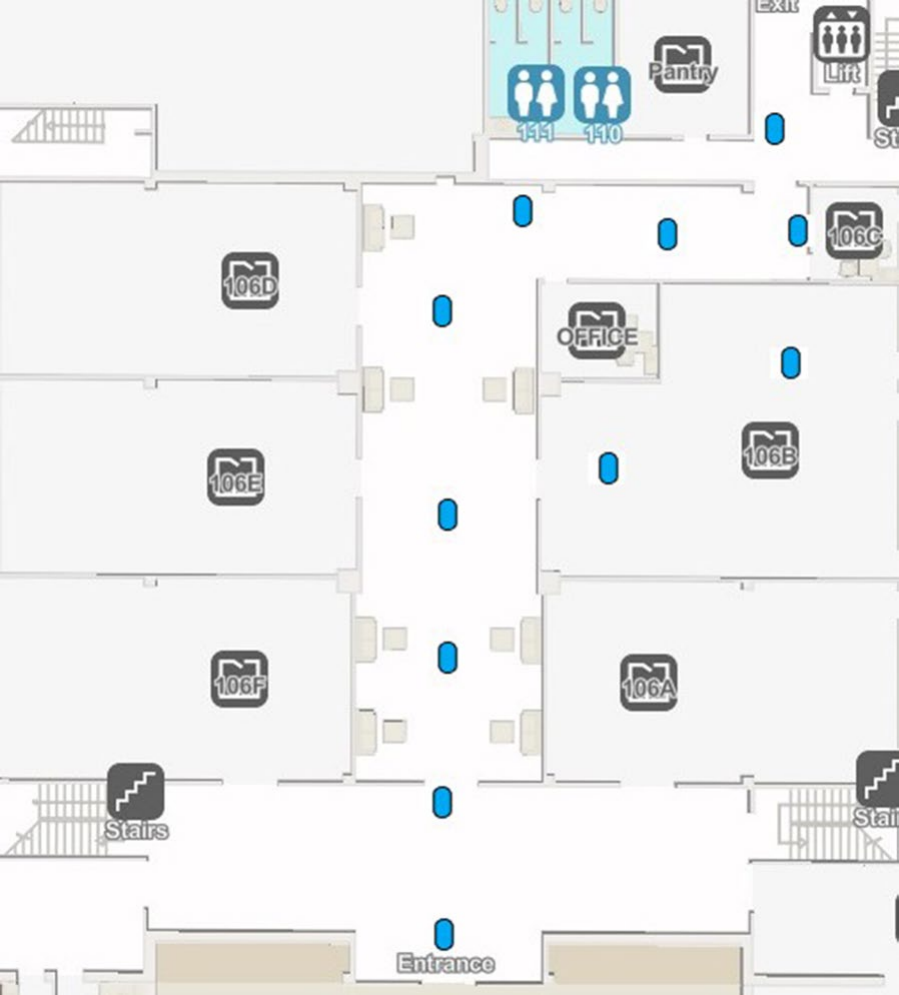
Distribution of beacons over testing environment

Beacons: The BLE beacon infrastructure was built using the beacons supplied by Steerpath. The BLE beacons are fixed in the walls within a height of 2.5 m. Figure 9 shows the distribution of BLE beacons in the selected indoor area. The distance of separation between each beacon is maximum of 8 m.The hardware specifications of the BLE beacons are provided in Table 3.

**Table 3:** Hardware specification of BLE beacons

Model	Minew i3 robust smart beacon
Operating frequency	2.4 GHz (Bluetooth 4.0)
Transmission power	0 dBm output power
Transmission range	up to 50 m
Time interval	350 ms

#### Android application

The Android application is configured as described earlier in CamNav and QRNav to compare the three systems. However, BLE-based application does not require a server application to estimate the location of the user. The positioning module responsible for location estimation is implemented in the Android application. The Android application receives the signal from beacons and RSSI of the signals are processed for further position estimation task. In addition to the positioning module, Android application consists of an indoor map, navigation module for providing navigation instructions and an android text to speech module for speech synthesis.

## Evaluations and results

**Fig. 10:**
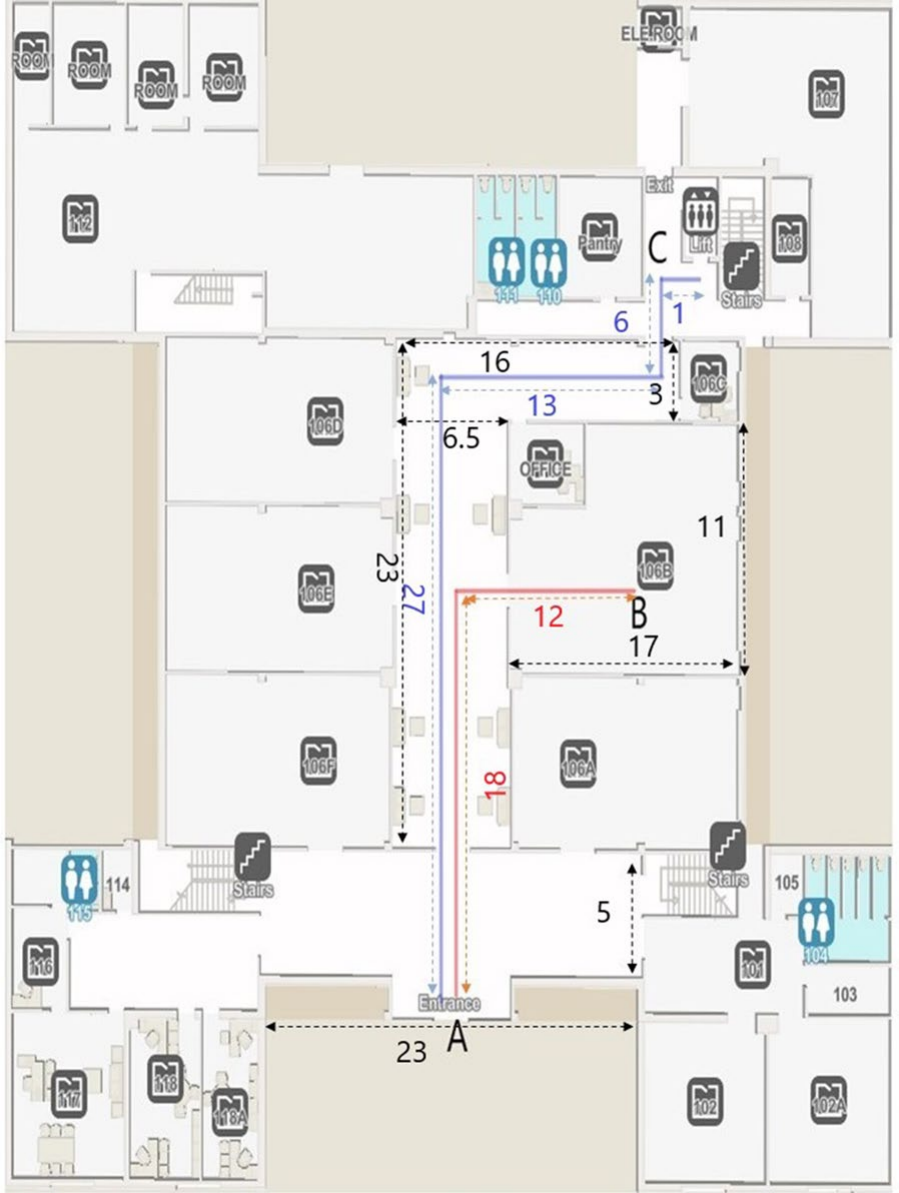
Floor plan of the ground floor of building B09 (All the dimension are given in meters. Red lines and blue lines indicates the navigation routes, while black lines indicate the dimension of rooms and corridors

We evaluated the performance of CamNav, QRNav and BLE beacons based navigation system in a real-world environment. This study was approved by the Qatar University Institutional Review Board. The approval number is QU-IRB 1174-EA/19. The evaluation experiments were carried out on the ground floor of the ‘B09’ building of Qatar University. Figure 10 illustrates the floor plan of the building ‘B09’ (ground floor).

We selected 10 people including 8 females and 2 males to evaluate the navigation systems in real-time. The age of participants is ranged from 22 to 39 (Mean = 29.30, standard deviation (SD) = 4.90). Since the participants were sighted, during experiments we made them wear blindfolds.

The blindfolded participants were asked to walk from the entrance door of the B09 building to two specific points of interest in the B09 building. Each participant has to walk from point A to B (Red line in the floor plan, distance = 30 m) and A to C (Blue line in the floor plan, distance = 47 m) using the three navigation systems separately. We recorded all the walking experiments using a video camera for further assessments.

To evaluate the performance of the systems and compare their merits and demerits, we mainly focused on computing the time required for the navigation using each system, error committed by the user in each navigation trail while using different systems and users’ feedback about the three systems.

### Navigation analysis

In this section, a study was conducted to examine the performance of the navigation systems in terms of time taken for traveling and errors that occurred during navigation. Ten blindfolded participants including 8 females and 2 males were asked to walk from point A to B and point A to C (shown in Fig. 10) using three navigation systems separately. All together each participant has to do six navigation experiments which include navigation through two routes (A to B and A to C) by using each of the three navigation systems separately.

For CamNav and QRNav systems, a Samsung Galaxy S7 smartphone was employed for running the android application. The Wi-Fi network of Qatar university was utilized as a medium for sending the images from the Android application to the server. On the server-side, an Asus ROG edition laptop with 24 GB ram and Nvidia GTX 1060 GPU was utilized for processing the captured images. BLE beacon-based system was directly implemented in the Samsung Galaxy S7 smartphone. Eleven BLE beacons are used to cover the whole navigation route. The positioning and hardware specifications of the beacons are detailed in the system overview section. Figure 8 shows the distribution of beacons over the experimental area.

Two metrics; time taken for navigation and error occurred during navigation in terms of the number of steps are considered in this navigation analysis to compare the performance of CamNav, QRNav, and BLE based system. The procedure followed for conducting the experiment is detailed as follows. Eyes of each participant are covered with a cloth prior to the navigation experiment. The details, as well as name of the source and destination of navigation routes, are not revealed to participants since they are familiar with the indoor areas of the building where the experiment is conducted. A participant is randomly called from 10 selected participants and asked to do the experiment in an order such that, walk from point A to B and A to C in two different trails using BLE application initially. Then we made the same participant repeat the experiment for two routes using the CamNav system followed by the QRNav system. The same procedure was repeated for the remaining nine participants. During each navigation trail, the time taken for navigation was measured using a stopwatch timer. Moreover, the error occurred during the navigation trail was measured manually in terms of the number of steps. In order to measure the error occurred during navigation, we considered some predefined points in the navigation route as location reference points. We considered four reference points in route 1 and seven reference points in route 2. The error occurred during navigation is computed as the sum of the number of steps the user is behind or ahead with respect to each predefined location reference points in the navigation route during the navigation by using each navigation system. The average time (in seconds) required by each navigation system for each route was displayed in Table  4. Route 1 represents point A to B and route 2 represents A to C.

**Table 4:** Average navigation time (standard deviation in brackets)

Systems	Average navigation time in seconds	
	Route 1	Route 2
CamNav	123.2 (11.66)	197.6 (18.31)
QRNav	117.2 (19.22)	204.1(16.33)
BLE APP	106.8 (16.88)	174.3 (23.19)

The average navigation time taken by participants while using BLE based system was comparatively less compared to the other two systems, CamNav and QRNav. The average time taken by the BLE based system for the shorter route (route A to B) and longer route (route A to C) was 106.8 s with a standard deviation of 16.88 and 174.3 s with a standard deviation of 23.19 respectively. While comparing with BLE based system, the CamNav system took 17 s and 23 s more average time in routes 1 and 2 respectively. The average time taken by the QRNav system is almost similar to the CamNav system. The main reason for the difference in average time while using different systems are, the client-server communication used in CamNav and QRNav consumes time, but less than 2 s for processing each image. Moreover, CamNav and QRNav provide abundant information regarding the current location and surroundings such as information about doors or walls. So the user has to wait for some seconds to hear and understand all the instructions. But the BLE beacon-based system will provide only the limited instructions to reach the destination. While using the QRNav and CamNav systems, we have noted that the results are affected by the walking speed of the user. This is because when the user is walking at a higher speed, the chance of getting blurry pictures is higher. In fact, the average speed of people with visual impairments varies according to their prior knowledge of the area they are walking in. BLE beacons based system enabled the user to walk a little bit fast and thereby reduced the navigation time. BLE beacons based system is able to provide real-time navigation service, while QRNav and CamNav are experiencing a small delay up to 2 s.

The average error obtained in each system is illustrated in Table 5.

**Table 5:** Average error in terms of the number of steps (Standard deviation in brackets)

Systems	Average error	
	Route 1	Route 2
CamNav	3.1 (0.56)	6.1 (1.10)
QRNav	3.3 (0.48)	5.5 (0.84)
BLE APP	4.3 (0.94)	8.7 (1.33)

The average errors in terms of the number of steps were less in CamNav and QRNav compared to BLE based system. The average error occurred in CamNav was 3.1 with a standard deviation of 0.56 and 6.1 with a standard deviation of 1.10 respectively for routes 1 and 2. In the QRNav system similar average value was obtained, 3.3 with a standard deviation of 0.48 in route 1 and 5.5 with a standard deviation of 0.84 in route 2. It proved that CamNav and QRNav are more accurate in providing reliable navigation services for users. The accuracy of the BLE beacon-based system was around 2-3 meters that raised conflicts about the user’s position while moving through the junctions. This conflict in position estimation generated uncertainty in provided navigation instructions and thereby misguided the user.

Navigation efficiency index (NEI) [62] was included to evaluate the navigation performance of the systems.NEI is defined as the ratio of actual traveled path’s distance to optimal path’s distance between source and destination. The NEI obtained in each system are displayed in Table 6. The obtained NEI in each system are acceptable and it displayed the effectiveness of navigation instructions provided by each system.

**Table 6:** Navigation efficiency index recorded in the systems

System	NEI
CamNav	1.010
QRNav	0.996
BLE APP	1.019

**Fig. 11:**
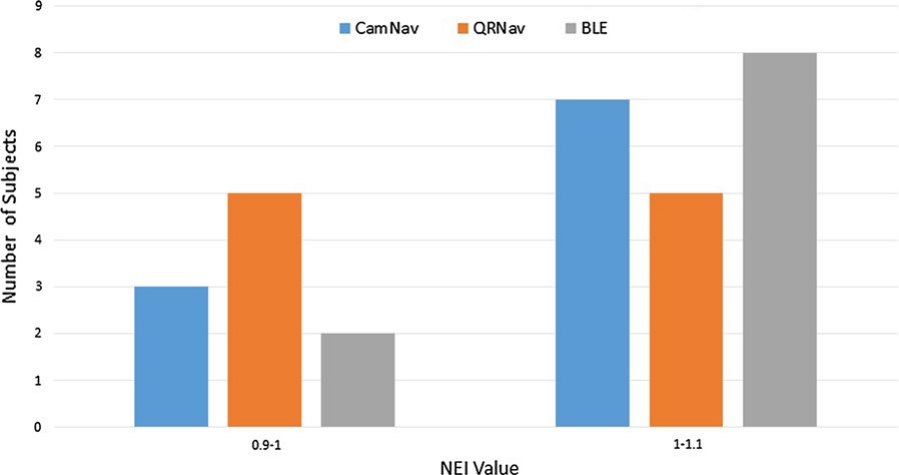
Distribution of navigation efficiency index

Distribution of NEI values among participants of navigation experiment for each system are shown in Fig. 11.

### Qualitative analysis

In order to examine the effectiveness of three systems, we prepared a questionnaire for the people who participated in the experiments. After completing the real-time experiments, we asked the participants to fill out the questionnaire. The questionnaire contains two sections. In the initial section, the participants are asked to rate the main functionalities (Navigation path suggestion, Location estimation, and Speech instructions) of the navigation systems using a Likert scale of points 1 (very bad) to 5 (excellent). The average rating of the functionalities is shown in Table 7.

**Table 7:** Rating of functionalities in the systems

Functionalities	Navigation systems		
	CamNav	QRNav	BLE APP
Navigation path suggestion	4.3	4.5	3.5
Location estimation	4.3	4.8	3.8
Speech instructions	4	4	4

“Related work” section of the questionnaire is dedicated to evaluating the satisfaction of the participants, the effectiveness of three navigation systems. In order to evaluate the satisfaction of the participants and effectiveness, we adopted a popular method known as the System Usability Scale (SUS) which has been commonly used in the literature for the usability evaluation task for various human-computer interaction systems. SUS is a simple, “quick and dirty” reliable tool to evaluate the usability of the systems. The high reliability, as well as the validity of the SUS tool, made it popular among the human-computer interaction community. The SUS tool consists of ten questions. Out of the ten questions, five (questions having odd serial numbers) are positive items and the remaining five (questions having even serial numbers) are negative items. The questions of the SUS questionnaire tool are displayed in Table 8.

**Table 8:** SUS questionnaire tool

I think that I would like to use this system frequently	
Found the system unnecessarily complex	
I thought the system was easy to use	
I think that I would need the support of a technical person to be able to use this system	
I found the various functions in this system were well integrated	
I thought there was too much inconsistency in this system	
I would imagine that most people would learn to use this system very quickly	
I found the system very cumbersome to use	
I felt very confident using the system	
I needed to learn a lot of things before I could get going with this system	

The participants are asked to respond and record their agreement for each question with a 5 point Likert scale where 1 means strongly disagree and 5 means strongly agree to the respective statements. Once the participants record their rating in terms of values, following equation is utilized to compute the overall SUS score of the system.

5 $$\begin{aligned} SUS\;score = 2.5\times \left[\displaystyle \sum \limits _{n=1}^5 (R_{2n-1}- 1) + (5-R_{2n})\right] \end{aligned}$$

In Eq. (1) Ri means the rating value (1 to 5) given for the ith question by the participant and n is the serial number of question. Overall SUS score calculation follows a simple procedure. For each odd number question or positive item, 1 will be subtracted from the rating value given by the participant. For each even number questions or negative item, the rating value given by the participants is subtracted from a constant value 5. Once the above mentioned subtraction operations are done, the obtained final values for each question are added and sum of these values are multiplied by 2.5. The overall SUS score ranges between 0 to 100, where higher value indicate superior performance. The average SUS score obtained for each system , standard deviation and Cronbach’s alpha of the SUS questionnaire for each system are displayed in Table 9. The Cronbach’s alpha is a commonly used statistical reliability analysis method to measure the internal consistency of questionnaire.

**Table 9:** System usability scores

	Navigation systems		
	CamNav	QRNav	BLE APP
Average overall SUS score	84.3	88	76.75
Standard deviation of SUS score	7.79	5.98	7.64
Cronbach’s alpha	0.73	0.61	0.61

The average usability score based on the SUS questionnaire for the CamNav system is 84.5 with a standard deviation of 7.79. QRNav obtained an average score of 88 with a standard deviation of 5.98 and which is the highest among all the three systems. BLE based application obtained the least average score (76.75 with a standard deviation of 7.64). While comparing these three systems based on the usability score, QRNav and CamNav displayed superior performance. Despite the fact that the BLE app obtained the least score, the SUS score obtained for each system including the BLE app are acceptable since the values are above 70. The Cronbach alpha which indicates the reliability of scores obtained in the questionnaire is 0.73 for SUS questionnaire in the CamNav system. Incase of QRNav and BLE based application the Cronbach alpha is 0.61. The obtained Cronbach alpha value shows the strong reliability for the SUS tool used in usability evaluation.

## Discussion

The results obtained from the real-time experiments and feedback from the participants show the effectiveness of the CamNav and QRNav systems over the BLE beacon-based navigation system. All the participants reached their destination with minimal external assistance. Some of the participants relied on external assistance while passing through the junctions in the navigation route. The external assistance was required in the junctions, when the user is turning to his right or left side. Instead of turning 90°, sometimes, they are turning more than that and it will lead to a condition where the user is deviating from the original path. While using BLE based application, it has noted that the is user bumped to the wall or door sometimes. But this issue was not present in the QRNav and CamNav systems. Because the QRNav is able to detect the doors or walls with the help of QR codes attached to it. CamNav is trained to recognize the walls and door areas in the indoor region.

It is clear from the user’s feedback that the participants found that Android application was easy to use. Minimal training is only required for using the Android application. Any user with minimum knowledge of smartphone can use this application for navigation. In case of QRNav and CamNav user has to keep the mobile straight or down for location recognition. Some user has found that it is difficult to position the smartphone in a fixed direction for long time. The majority of the participants said that they will suggest the CamNav or QRNav system for their visually impaired friend or relatives. However the current version of CamNav and QRNav provides information about the doors in the navigation route, the participants suggested adding a module to detect and recognize the static (e.g. table, chair, etc.) and dynamic (e.g. people) objects or obstacles to ensure safe navigation.

The participants for the field test has been randomly selected and the criteria for selecting the participants was minimum knowledge of smartphone and English language understanding. The selected participants are not having any physical disability as well as hearing disability. since they have to walk properly by closing eyes with the help of voice instructions. To best of our knowledge , the participant selection has not adversely effected the result. During experiments a minimal help was provide for blindfolded users in order to preserve their safety. For each participants, all six navigation trails was conducted on a same day, with providing proper rest between each trail. All of the three system are tested by same group of 10 participants. The device and instruments used for experiments were same for all participants during each trail. Moreover same tool (navigation analysis and usability evaluation using SUS tool) was used for assessing the performance of three systems. Despite the fact that the participants are not visually impaired, we believe the obtained result are enough to compare the three different approaches for indoor navigation.

While comparing three different approaches we found that the QR code recognition is very quick and precise. It doesn’t require powerful processors or devices to carry out the QR code decoding process. On other hand CNN based scene recognition is effective for identifying the doors or walls or other static obstacle in the navigation path. Same can be achieved in QR code system, but it requires pasting the QR codes in walls and doors. And it is not practically possible to paste QR codes in all walls and doors since it is not aesthetically pleasing for other people with normal vision. On the other hand CNN recognition may fail in locations with similar appearance. The BLE beacons are not much accurate to estimate the exact position of the user. But it can be used to identify the rooms or corridor where the user is present. While navigating through straight paths in corridors BLE beacons are effective for getting real time position of the user. All of the three approaches have merits as well as limitations. But three approaches can be integrated to develop a hybrid navigation system for people with visual impairments where merit of one system will compensate the limitation of other such as QR code recognition or BLE beacons positioning can solve the issue of recognizing locations with similar appearance using CNN model.

We already provided the comparison of some existing indoor navigation solutions for people with VI in Table 2. With respect to Table 2 , here we provide the comparison of three developed systems in Table 10 while considering the same attributes used in Table 2.

**Table 10:** Comparison of developed indoor navigation systems

Reference	Technology	System	Techniques	Tested by	Test	Feedback	Accuracy	Remarks
CamNav	Computer vision	Smartphone, laptop	CNN based scene recognition	Blindfolded	Error occurred during navigation and navigation time	Voice	Medium	(−) Hard to differentiate indoor locations with similar appearance
QRNav	Computer vision	Smartphone, laptop, QR codes	QR code recognition	Blindfolded	Error occurred during navigation and navigation time	Voice	Medium	(−) Requires large amount of QR codes for safe navigation
BLE base system	BLE	Beacons and smartphone	Fingerprinting and trilateration	Blindfolded	Error occurred during navigation and navigation time	Voice	Low	(−) Relatively low accuracy resulted in navigation errors

## Conclusion

In this article, we developed three indoor navigation systems for people with visual impairments. The proposed systems utilize different technologies, CNN based scene recognition, QR code recognition and BLE based positioning to navigate people with visual impairments. The three systems has been implemented and assessed in indoor environment. BLE based system took less time for navigation, while QRNav and CamNav showed better performance while considering the average error obtained in each system and system usability scores.

Three systems was tested on blindfolded people in a real-time environment. The average error obtained in CamNav is 3.1 steps and 6.1 steps with a standard deviation of 0.56 and 1.10 respectively in route 1 and route 2. The average error was highest in BLE based application with a value 4.3 steps and 8.7 steps with standard deviation 0.94 and 1.33 in routes 1 and 2 respectively. In usability experiments , QRNav obtained highest system usability score of 88, CamNav got a score of 84.3 and BLE based system got an average score of 76.75. Indoor scene recognition and QR code recognition are affected by the walking speed of the user. Embedding a obstacle detection sensor along with navigation system can ensure the safety of user during the presence of mobile obstacle. In the future, we will consider integrating QR code recognition in CamNav system to cope with the identification of location which is similar in appearance. The future version of CamNav will address most of the requirements suggested by the users who participated in the real-time evaluation.

## Data Availability

Up on reasonable request, the corresponding author will make the datasets available.

## References

[1] Fallah N, Apostolopoulos I, Bekris K, Folmer E (2013). Indoor human navigation systems: a survey. Interact Comput..

[2] Maghdid HS, Lami IA, Ghafoor KZ, Lloret J (2016). Seamless outdoors-indoors localization solutions on smartphones: implementation and challenges. ACM Comput Surv (CSUR)..

[3] Koyuncu H, Yang SH (2010). A survey of indoor positioning and object locating systems. IJCSNS Int J Comput Sci Netw Secur..

[4] Fangmeyer JR JA, Rosales CV, Rodríguez DM, Pinero RFB. Evolution of indoor positioning technologies: a survey. 2018.

[5] Ben-Afia A, Deambrogio L, Salós D, Escher AC, Macabiau C, Soulier L (2014). Review and classification of vision-based localisation techniques in unknown environments. IET Radar Sonar Navig..

[6] Jeon KE, She J, Soonsawad P, Ng PC (2018). BLE beacons for Internet of Things applications: survey, challenges, and opportunities. IEEE Internet Things J..

[7] Chang K (2014). Bluetooth: a viable solution for IoT? [Industry Perspectives]. IEEE Wireless Commun..

[8] Yin H, Jiao X, Chai Y, Fang B (2015). Scene classification based on single-layer SAE and SVM. Expert Syst Appl..

[9] Zhou B, Lapedriza A, Khosla A, Oliva A, Torralba A (2017). Places: A 10 million image database for scene recognition. IEEE Trans Pattern Anal Mach Intell..

[10] Hightower J, Borriello G (2001). Location systems for ubiquitous computing. Computer..

[11] Iglesias HJP, Barral V, Escudero CJ. Indoor person localization system through RSSI Bluetooth fingerprinting. In: 2012 19th international conference on systems, signals and image processing (IWSSIP). New York: IEEE; 2012; p. 40–43.

[12] Clark BK, Winkler EA, Brakenridge CL, Trost SG, Healy GN (2018). Using Bluetooth proximity sensing to determine where office workers spend time at work. PloS ONE..

[13] Zandbergen PA, Barbeau SJ (2011). Positional accuracy of assisted GPS data from high-sensitivity GPS-enabled mobile phones. J Navig..

[14] Lindeberg T (2012). Scale invariant feature transform. Scholarpedia..

[15] Speeded-Up Robust Features (SURF). Computer vision and image understanding. 2008;110(3):346 – 359. Similarity Matching in Computer Vision and Multimedia. http://www.sciencedirect.com/science/article/pii/S1077314207001555.

[16] Wang Hai, Zhang Shuwu (2011). Evaluation of Global Descriptors for Large Scale Image Retrieval. Image Analysis and Processing – ICIAP 2011.

[17] LeCun Y, Bengio Y, Hinton G (2015). Deep learning.. Nature..

[18] Lee YH, Medioni G, Agapito L, Bronstein MM, Rother C (2015). Wearable RGBD indoor navigation system for the blind. Computer vision—ECCV 2014 workshops.

[19] Kamisaka D, Muramatsu S, Iwamoto T, Yokoyama H (2011). Design and implementation of pedestrian dead reckoning system on a mobile phone. IEICE Trans Inf Syst..

[20] Ban R, Kaji K, Hiroi K, Kawaguchi N. Indoor positioning method integrating pedestrian Dead Reckoning with magnetic field and WiFi fingerprints. In: 2015 Eighth international conference on mobile computing and ubiquitous networking (ICMU); 2015. p. 167–72.

[21] Woodman OJ. An introduction to inertial navigation. University of Cambridge, Computer Laboratory; 2007. UCAM-CL-TR-696. https://www.cl.cam.ac.uk/techreports/UCAM-CL-TR-696.pdf.

[22] Bouet M, dos Santos AL. RFID tags: Positioning principles and localization techniques. In: 2008 1st IFIP wireless days; 2008; p. 1–5.

[23] Fu Q, Retscher G (2009). Active RFID trilateration and location fingerprinting based on RSSI for pedestrian navigation. J Navig..

[24] He S, Chan SG (2016). Wi-Fi fingerprint-based indoor positioning: recent advances and comparisons. IEEE Commun Surv Tutor.

[25] Farid Z, Nordin R, Ismail M (2013). Recent advances in wireless indoor localization techniques and system. J Comput Netw Commun..

[26] Orujov F, Maskeliūnas R, Damaševičius R, Wei W, Li Y (2018). Smartphone based intelligent indoor positioning using fuzzy logic. Future Gener Comput Syst..

[27] Do Trong-Hop, Yoo Myungsik (2016). An in-Depth Survey of Visible Light Communication Based Positioning Systems. Sensors.

[28] Alarifi Abdulrahman, Al-Salman AbdulMalik, Alsaleh Mansour, Alnafessah Ahmad, Al-Hadhrami Suheer, Al-Ammar Mai, Al-Khalifa Hend (2016). Ultra Wideband Indoor Positioning Technologies: Analysis and Recent Advances. Sensors.

[29] Tian Y, Yang X, Yi C, Arditi A (2013). Toward a computer vision-based wayfinding aid for blind persons to access unfamiliar indoor environments. Mach Vis Appl..

[30] Deniz O, Paton J, Salido J, Bueno G, Ramanan J. A vision-based localization algorithm for an indoor navigation app. In: 2014 Eighth international conference on next generation mobile apps, services and technologies. New York: IEEE; 2014. p. 7–12.

[31] CANNY JOHN (1987). A Computational Approach to Edge Detection. Readings in Computer Vision.

[32] Garcia G, Nahapetian A. Wearable computing for image-based indoor navigation of the visually impaired. In: Proceedings of the conference on Wireless Health. New York: ACM; 2015. p. 17.

[33] Manlises C, Yumang A, Marcelo M, Adriano A, Reyes J. Indoor navigation system based on computer vision using CAMShift and D* algorithm for visually impaired. In: 2016 6th IEEE international conference on control system, computing and engineering (ICCSCE). New York: IEEE; 2016; p. 481–4.

[34] Tian Yingli, Yang Xiaodong, Arditi Aries (2010). Computer Vision-Based Door Detection for Accessibility of Unfamiliar Environments to Blind Persons. Lecture Notes in Computer Science.

[35] Costa P, Fernandes H, Martins P, Barroso J, Hadjileontiadis LJ (2012). Obstacle detection using stereo imaging to assist the navigation of visually impaired people. Procedia Comput Sci..

[36] Lee YH, Medioni G (2016). RGB-D camera based wearable navigation system for the visually impaired. Computer Vis Image Understand..

[37] Li B, Muñoz JP, Rong X, Chen Q, Xiao J, Tian Y (2018). Vision-based mobile indoor assistive navigation aid for blind people. IEEE Trans Mob Comput..

[38] Kanwal N, Bostanci E, Currie K, Clark AF. A navigation system for the visually impaired: a fusion of vision and depth sensor. Appl Bionics Biomech. 2015; 2015. 10.1155/2015/479857 PMC4745441 27057135

[39] Idrees A, Iqbal Z, Ishfaq M. An efficient indoor navigation technique to find optimal route for blinds using QR codes. In: 2015 IEEE 10th conference on industrial electronics and applications (ICIEA). New York: IEEE; 2015; p. 690–5.

[40] Al-Khalifa S, Al-Razgan M (2016). Ebsar: indoor guidance for the visually impaired. Comput Electr Eng..

[41] Gude R, Østerby M, Soltveit S (2013). Blind navigation and object recognition.

[42] Legge GE, Beckmann PJ, Tjan BS, Havey G, Kramer K, Rolkosky D (2013). Indoor navigation by people with visual impairment using a digital sign system. PloS ONE..

[43] Zeb A, Ullah S, Rabbi I. Indoor vision-based auditory assistance for blind people in semi controlled environments. In: 2014 4th international conference on image processing theory, tools and applications (IPTA). New York: IEEE; 2014. p. 1–6.

[44] Huang YC, Ruan SJ, Christen O, Naroska E. A wearable indoor locating system based on visual marker recognition for people with visual impairment. In: 2016 IEEE 5th global conference on consumer electronics. New York: IEEE; 2016. p. 1–2.

[45] Raj CR, Tolety S, Immaculate C. QR code based navigation system for closed building using smart phones. In: 2013 International mutli-conference on automation, computing, communication, control and compressed sensing (iMac4s). New York: IEEE; 2013. p. 641–4.

[46] Chuang CH, Hsieh JW, Fan KC. A smart handheld device navigation system based on detecting visual code. In: 2013 international conference on machine learning and cybernetics. vol. 3. New York: IEEE; 2013; p. 1407–12.

[47] Rituerto A, Fusco G, Coughlan JM. Towards a sign-based indoor navigation system for people with visual impairments. In: Proceedings of the 18th international ACM SIGACCESS conference on computers and accessibility. New York: ACM; 2016. p. 287–8. 10.1145/2982142.2982202 PMC5714555 29214242

[48] Ahmetovic D, Gleason C, Ruan C, Kitani K, Takagi H, Asakawa C. NavCog: a navigational cognitive assistant for the blind. In: Proceedings of the 18th international conference on human-computer interaction with mobile devices and services. New York: ACM; 2016. p. 90–9.

[49] Kim JE, Bessho M, Kobayashi S, Koshizuka N, Sakamura K. Navigating visually impaired travelers in a large train station using smartphone and Bluetooth low energy. In: Proceedings of the 31st annual ACM symposium on applied computing. New York: ACM; 2016. p. 604–11.

[50] Cheraghi SA, Namboodiri V, Walker L. GuideBeacon: Beacon-based indoor wayfinding for the blind, visually impaired, and disoriented. In: 2017 IEEE international conference on pervasive computing and communications (PerCom). New York: IEEE; 2017. p. 121–30.

[51] Bilgi S, Ozturk O, Gulnerman AG. Navigation system for blind, hearing and visually impaired people in ITU Ayazaga campus. In: 2017 international conference on computing networking and informatics (ICCNI). New York: IEEE; 2017. p. 1–5.

[52] Duarte Karen, Cecílio José, Furtado Pedro (2015). Easily Guiding of Blind: Providing Information and Navigation - SmartNav. Lecture Notes of the Institute for Computer Sciences, Social Informatics and Telecommunications Engineering.

[53] Murata M, Ahmetovic D, Sato D, Takagi H, Kitani KM, Asakawa C (2019). Smartphone-based localization for blind navigation in building-scale indoor environments. Pervas Mob Comput..

[54] AL-Madani Basem, Orujov Farid, Maskeliūnas Rytis, Damaševičius Robertas, Venčkauskas Algimantas (2019). Fuzzy Logic Type-2 Based Wireless Indoor Localization System for Navigation of Visually Impaired People in Buildings. Sensors.

[55] Snyder B, Bosanac D, Davies R. Introduction to apache activemq. Active MQ in action. 2017. p. 6–16.

[56] Abadi M, Agarwal A, Barham P, Brevdo E, Chen Z, Citro C, et al. Tensorflow: Large-scale machine learning on heterogeneous distributed systems. arXiv preprint arXiv:160304467. 2016.

[57] CamNav-dataset. https://github.com/akarkar/CamNav-dataset. Accessed 30 Apr 2019.

[58] Mapbox. https://www.mapbox.com. Accessed 6 Nov 2019.

[59] Zxing. http://code.google.com/p/zxing/. Accessed 4 Nov 2019.

[60] Zbar bar code reader. http://zbar.sourceforge.net/. Accessed 25 Apr 2019.

[61] Shchekotov M. Indoor localization method based on Wi-Fi trilateration technique. In: Proceeding of the 16th conference of fruct association; 2014. p. 177–9.

[62] Ganz A, Schafer J, Gandhi S, Puleo E, Wilson C, Robertson M (2012). PERCEPT indoor navigation system for the blind and visually impaired: architecture and experimentation. Int J Telemed Appl..

